# Exploring the Individual Bacterial Microbiota of Questing *Ixodes ricinus* Nymphs

**DOI:** 10.3390/microorganisms9071526

**Published:** 2021-07-17

**Authors:** Aurélien Alafaci, Alexandre Crépin, Sabine Beaubert, Jean-Marc Berjeaud, Vincent Delafont, Julien Verdon

**Affiliations:** Laboratoire Ecologie & Biologie des Interactions, UMR CNRS 7267, Université de Poitiers, 1 Rue Georges Bonnet, TSA 51106, CEDEX 9, 86073 Poitiers, France; aurelien.alafaci@univ-poitiers.fr (A.A.); alexandre.crepin@univ-poitiers.fr (A.C.); sabinebbt@gmail.com (S.B.); jean-marc.berjeaud@univ-poitiers.fr (J.-M.B.); vincent.delafont@univ-poitiers.fr (V.D.)

**Keywords:** *Borrelia*, questing ticks, metabarcoding, microbiota, *Ixodes*, *Borreliella*

## Abstract

*Ixodes ricinus* is the most common hard tick species in Europe and an important vector of pathogens of human and animal health concerns. The rise of high-throughput sequencing has facilitated the identification of many tick-borne pathogens and, more globally, of various microbiota members depending on the scale of concern. In this study, we aimed to assess the bacterial diversity of individual *I. ricinus* questing nymphs collected in France using high-throughput 16S gene metabarcoding. From 180 dragging-collected nymphs, we identified more than 700 bacterial genera, of which about 20 are abundantly represented (>1% of total reads). Together with 136 other genera assigned, they constitute a core internal microbiota in this study. We also identified 20 individuals carrying *Borreliella*. The most abundant species is *B. afzelii*, known to be one of the bacteria responsible for Lyme disease in Europe. Co-detection of up to four *Borreliella* genospecies within the same individual has also been retrieved. The detection and co-detection rate of *Borreliella* in *I. ricinus* nymphs is high and raises the question of interactions between these bacteria and the communities constituting the internal microbiota.

## 1. Introduction

Ticks are blood-sucking arthropods known worldwide to be vectors of many pathogens for humans, as well as for domestic and wild animals [1]. Ticks ingestion of blood is completed by biting at each developmental stage to induce the moult [2]. During these meals, ticks can take up pathogens by feeding on an infected host or by co-feeding [3,4]. While feeding, an infected tick can also transmit pathogens with its saliva to a new host. Tick-borne pathogens (TBPs) are an integral part of the tick microbiome, along with commensals and symbiotic viruses, archaea, bacteria and eukaryotes [5]. However, until the beginning of 2011, this microbiome was largely unknown [6]. Over the last decade, many studies have focused on its description in several tick species throughout the world, at different stages of the life cycle, during questing or during interactions with various hosts [7,8,9,10,11,12,13,14]. The increasing amount of available data allows us to gain a clearer insight into the different microbial communities associated with ticks. However, a current lack of harmonization at the level of collection and/or analysis conditions still limits our understanding of both the composition of these communities and the relationships between these microorganisms [15].

The role played by the microbiome in the establishment and transmission of pathogens, as well as on tick biology, has received increasing attention in recent years [16,17]. Numerous maternally inherited bacteria have been described in ticks, although their functional roles are largely unknown [18]. Nonetheless, one (*Francisella* F-Om) has been shown to supply B vitamins and cofactors, counterbalancing the deficiency induced by a blood-only diet [19]. As obligate endosymbionts in most tick species, *Coxiella*-like are also suspected to be nutritional symbionts of ticks [20]. TBPs include viruses, bacteria and parasites responsible for several infections. Their presence may impact their host microbiome in the same way as others factors such as temperature [21], tick species, developmental stage, geographical origin [13,22], anatomical region and sex [23,24,25]. Although less studied, multiple and complex interactions between microorganisms take place, influencing the ability of TBPs to colonize the gut and their ability to be transmitted to a new host [26,27,28,29]. The composition of the microbiome is also a driver of gene expression and proteins production in ticks. It promotes the establishment of certain microorganisms such as the Lyme disease (LD)-causing spirochete *Borreliella burgdorferi* [30]. Interestingly, differences in the relative abundance of certain taxa were linked to the presence/absence of *B. burgdorferi,* whereas the spirochete load seems poorly affected by the internal bacterial composition [11]. Furthermore, DNA of several TBPs have regularly been detected within the same tick suggesting their possible coexistence and, in some cases, a proven co-infection [31,32,33,34,35]. These studies highlight the complexity of interactions among microbial populations within ticks that do not only involve pathogenic members. To date, the impact of those interactions between TBPs on human infections remains poorly understood [36,37]. Numerous factors modulate these interactions, one of those factors being the impact of co-infections in human health, raising difficulties to perform a diagnosis and to offer therapeutic solutions [38,39].

Most TBPs are transmitted during feeding by the bite of ticks belonging to the *Ixodidae* family [39]. Among the genera composing this family are the ticks of the genus *Ixodes* that vectorize *B. burgdorferi*, the LD spirochete. In the Northern Hemisphere, LD is the most common vector-borne disease, with approximately 30,000 cases reported to the Center for Disease Control and Prevention (this number does not reflect all cases of LD; recent estimates suggest that approximately 476,000 people may contract LD each year in the United States) and around 200,000 new cases in Western Europe [40,41]. In the Eastern United States, *B. burgdorferi* is primarily vectored by *I. scapularis*, while *I. pacificus* is the prime vector to humans on the Western coast [42]. In Western Europe, the principal tick vector is *I. ricinus,* while *I. persulcatus* predominates in the Eastern part of the continent and in Asia [43]. Within the *Borreliaceae* family, species responsible for LD are referred to as *B. burgdorferi sensu lato* and are not evenly distributed around the world. The global distribution is mainly dependent on the considered continent [44,45]. The most pathogenic species for humans in Northern America is *B. burgdorferi sensu stricto* (ss), whereas in Europe, *B. afzelii* and *B. garinii* are the main pathogenic species, even though cases associated with *B. burgdorferi* ss, *B. spielmanii* and *B. bavariensis* have been reported [45,46,47,48].

In this study, we aimed to unravel the bacterial diversity of individual *I. ricinus*. Questing nymphs were collected at four different sites in the former region Poitou-Charentes in France. The bacterial microbiota of individual nymphs was assessed using high throughput sequencing of the 16S rRNA coding gene sequence with a focus on TBPs, including *Borreliella* genospecies. We also investigated the existence of several pathogens in the microbiota of each collected tick.

## 2. Materials and Methods

### 2.1. Tick Sampling and Processing

Questing nymphs of *I. ricinus* were collected in June 2019 at four locations in Poitou-Charentes (France): Bignoux (46°36′24″ N, 0°28′14″ E), Lezay (46°14′40″ N, 0°3′30″ E), Hiesse (46°2′14″ N, 0°36′17″ E) and Tonnay-Charente (15°57′12″ N, 0°54′12″ W). All sampling sites are located within a radius of 67 km. Ticks were collected by a dragging protocol with a 1 m^2^ piece of white linen attached to a stick connected to a rope. Dragging on vegetation was carried out at each of the four sites on 20 transects of 10 m, each spaced from 10 to 15 m. Approximately 100 individuals were collected at each site, brought back to the laboratory in sterile 50 mL tubes and kept alive at room temperature. Each tick was examined under a Wild Heerbrugg M3 (Leica, Wetzlar, Germany) stereomicroscope using morphological identification criteria [49] before washing. Cuticular fauna, including bacteria and exogenous DNA, were eliminated following the bleach-based protocol described in detail in another study [50]. Briefly, ticks were bathed individually for 30 s in a commercial bleach solution diluted to a final concentration of 1%. Ticks were then rinsed three times in successive DNA-free water baths for 1 min. Immediately after the last bath, ticks were individually stored in sterile 1.5 mL tubes filled with 70% ethanol and stored at −20 °C prior to DNA extraction.

### 2.2. DNA Extraction

Forty-five *I. ricinus* nymphs per sampling site were individually crushed in liquid nitrogen using a mortar and pestle. To avoid cross-contamination between samples, the mortar and pestle were sterilized by washing with commercial bleach. After crushing, the tissues were transferred to sterile tubes for DNA extraction. The mortar and pestle were then rinsed with the first buffer of the DNA extraction kit to limit the loss of tissues. Negative crushing controls were carried out without ticks. DNA from all samples was then extracted using the DNeasy^®^ Blood&Tissue Kit (Qiagen, Hilden, Germany) following the manufacturer’s instructions. Negative DNA extraction controls were carried out using only kit reagents.

### 2.3. PCR Amplification and High-Throughput Sequencing

A 464-bp fragment covering variable regions V3–V4 of the bacterial 16S rRNA gene was PCR-amplified using the U341F (5′-CCTACGGGRSGCAGCAG-3′) [51] and Bakt_805R (5′-GACTACHVGGGTATCTAATCC-3′) [52] primer pair. These primers were selected for their high coverage on 16S rRNA gene sequences [53]. The PCR was performed using Q5, high-fidelity DNA Polymerase (New England Biolabs, Evry, France) together with Q5 reaction buffer (1X final), 0.5 µM of each primer, dNTP (0.2 mM each) and 5 µL of DNA in a final volume of 40 µL. The amplification was carried out as follows: an initial denaturation step at 98 °C for 5 min then denaturation at 98 °C for 30 s, annealing at 56 °C for 30 s, elongation at 72 °C for 30 s for a total of 30 cycles, followed by a final elongation step at 72 °C for 5 min. For each PCR run, negative (DNA-free water) and positive (DNA extracted as previously described from an overnight culture of *P. aeruginosa* PA14 cultured in LB medium at 37 °C with shaking at 180 rpm) template controls were included. The PCR products were subjected to 1% agarose gel electrophoresis and further purified using the NucleoSpin^®^ Gel and PCR Clean-up kits (Macherey-Nagel, Bethlehem, PA, USA). Mock purifications were carried out as negative controls for further analysis. DNA quantification was carried out using a Qubit^®^ 2.0 fluorometer (Invitrogen, Waltham, MA, USA). For all PCR amplicons, the library was constructed and sequenced by the genotyping and sequencing platform of the Paris Brain Institute (Paris, France; https://icm-institute.org/fr/ accessed on 30 November 2019) using Illumina MiSeq single-end 1 × 500-bp technology. All raw data were deposited in the Sequence Read Archive of the NCBI under the BioProject ID PRJNA727936.

### 2.4. Sequencing Data Processing

Sequencing results were analyzed using the QIIME 2 2017.4 software package [54]. The quality of reads was assessed and filtered using the DADA2 software implemented in QIIME 2 [55]. To do this, 15 bp were removed at the 5′ side of all amplicons and truncated at 400 bp. The final reads used for analysis were 385-bp long. Reads were grouped into exact Amplicon Sequence Variants (ASVs) using DADA2. ASVs taxonomic affiliations were determined using VSEARCH [56], along with the Silva 132 16S database (https://www.arb-silva.de/ accessed on 10 February 2020) with a minimum similarity threshold of 90%. Reads that failed to be classified within the bacteria kingdom were further characterized by comparison with the nr/nt database, using the BLAST algorithm [57] with a maximum e-value cutoff of 0.0001. Reads assigned to ticks or eukaryotic organisms were eliminated from the dataset. To ensure efficient dealing with potential contaminant sequences, a two-step process was implemented. Firstly, sequences that likely originated from kit contamination were filtered out using the decontam package in the R environment [58]. Then, filtered samples and all controls were clustered, along with all controls, using the Bray–Curtis dissimilarity index (Supplementary Materials Figure S1). Samples grouping with controls were completely excluded from all downstream analyses. The R software was used for the map creation (sp [59], rnaturalearth and rnaturalearthdata [60], maps [61]), relative abundance analysis (phyloseq [62], tidyverse [63]) and ranked abundance analysis (vegan [64], BiodiversityR [65]), LDA analysis (MASS [66]). For figures generated in R, the ggplot2 [67] package was used. Core microbiota graphical representations were achieved using Krona [68], and Venn diagrams were drawn using InteractiVenn [69].

### 2.5. Borreliella Amplicon Sequence Variants Analysis

Amplicon Sequence Variants assigned to the *Borreliella* genus were individually compared to the NCBI nr/nt database using megablast. 16S rRNA coding gene sequences from *Borreliella* reference strains were selected and used to construct a phylogeny. Reads from *Ralstonia insidiosa* CCBE 1007-18 and *Treponema* I8:G57 were also included as outgroups, the latest being selected because *Treponema* belongs to the same family as *Borreliella*. All reads were truncated to a final length of 335 nucleotides. Alignments were realized using the MUSCLE algorithm implemented in MEGA-X software [70]. Model Finder [71], implemented in the IQ-TREE software [72], was used to select the best phylogeny construction model. According to the Bayesian Information Criterion, the best fit model was the Kimura Two-Parameter substitution model allowing for a proportion of invariable sites (K2P+I). The phylogenetic tree was generated using IQ-TREE, and the node robustness was assessed using 1000 iterations of SH-like approximate likelihood ratio test [73] and 1000 iterations of conventional bootstraps. Itol v4 [74] was used for tree representation and formatting.

## 3. Results

### 3.1. Collection of Questing Ticks

**Figure 1:**
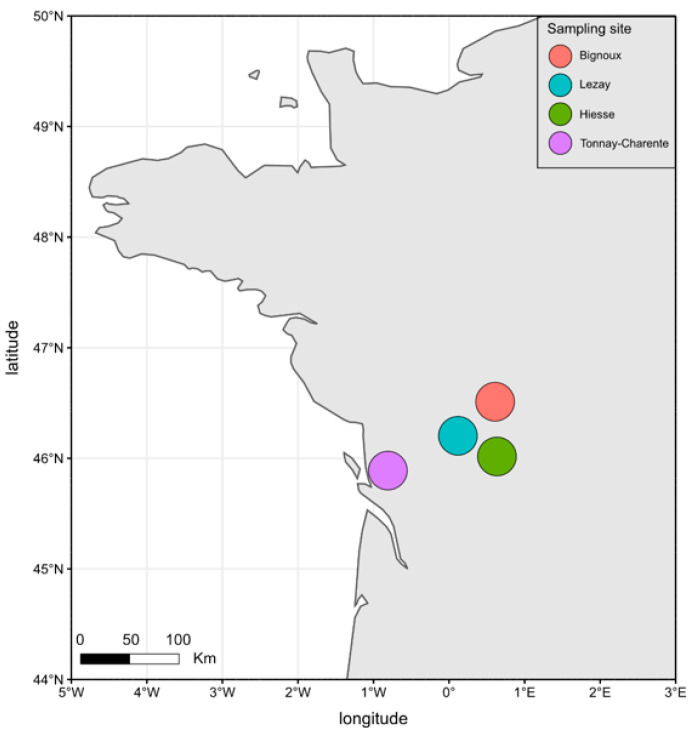
The sampling areas in Western France.

A total of 370 questing ticks were collected from four sites (Bignoux, Hiesse, Lezay and Tonnay-Charente) using a dragging protocol (Figure 1). Out of these 370 ticks, 93.0% (344/370) belonged to the species *Ixodes ricinus,* and 7.0% (26/370, only nymphs) belonged to the genus *Haemaphysalis* sp. Only *I. ricinus* ticks were considered in this study. Out of the 344 *I. ricinus* individuals, 315 were nymphs, and 29 were adults with 11 females and 18 males.

### 3.2. Sequencing of the Internal Bacterial Microbiota of Ticks

Sequencing was carried out on 180 randomly selected bleach-treated nymphs of *I. ricinus* (45 per sampling area) and 12 negative controls (four crushing controls, four DNA extraction controls and four purification controls). A total of 826,697 raw reads were generated, from which 370,095 high-quality reads were retained for further analyses. Out of these, 60,149 reads were assigned to ticks or eukaryotic organisms and were further discarded. As nymphal ticks are low biomass arthropods [75,76], the number of generated reads may be low on average, and variations in the read numbers can thus exist among samples. Moreover, such low biomass samples can be critically impacted by contamination originating from the various kits used to produce amplicons [77]. After potential contaminants sequences exclusion, five samples were excluded from the dataset, leaving 203,857 reads distributed among 175 individual nymphs. Alpha rarefaction analyses suggested that the sequencing effort was sufficient to capture most of the bacterial diversity of individual nymphs’ microbiota (Supplementary Materials Figure S2). To estimate the bacterial diversity present in the dataset, alpha diversity indexes (Shannon diversity index and Shannon evenness index), as well as ASVs diversity, were calculated for each sampling site (Table 1, Supplementary Materials Figure S3).

In terms of richness and diversity, the Shannon diversity index (H) highlighted the presence of many taxa with well-balanced abundances for each sampling site. When comparing sampling sites, the H index did not differ drastically, indicating few discrepancies in richness between samples retrieved from these sites. Concerning the bacterial taxa repartition at each site, the Shannon evenness index (E) indicated a fairly even abundance of all taxa rather than a strong dominance of certain taxa. The sampling site, therefore, had little impact on the degree of bacterial diversity hosted by *I. ricinus* nymphs.

### 3.3. Taxonomic Diversity in Nymphs

**Figure 2:**
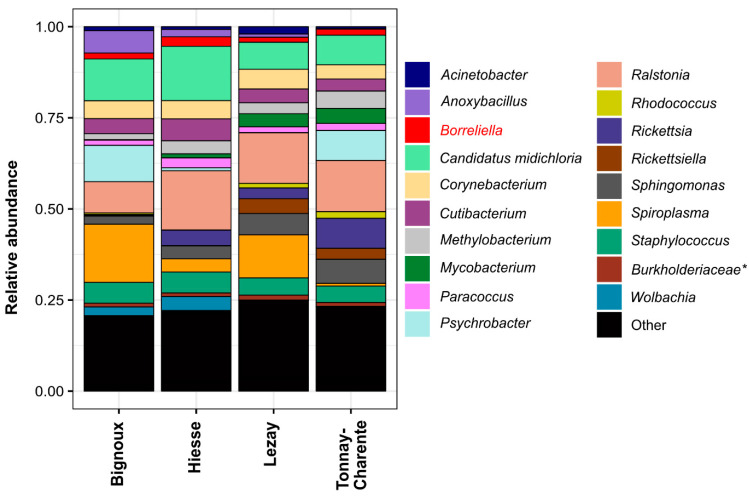
Relative abundance bar plot of the 20 most commonly detected taxonomic units grouped by sampling site. Taxa were classified at the genus level whenever possible. Each column represents grouped samples from a sampling site. Genera were represented depending on the overall reads number. The category « other » contains all taxa that do not belong to the 20 most abundant. *Borreliella* is in red because a focus on this genus is made later in the article. *: Assignment at the family level only because it was not feasible to have a more detailed assignment.

For further analyses, all ASVs were grouped together according to their taxonomy. All samples were also visualized according to their sampling site. The twenty most abundant taxa found in the dataset were first selected. They represent 73.27% of all reads (149,385/203,857 reads), and their abundance range from 13.17% for *Ralstonia* to 1.02% for *Methylobacterium* (Supplementary Materials Table S1). Their relative abundance per site was represented as a bar plot (Figure 2).

Of these genera, the two most abundant were *Ralstonia* (26,761 reads) and *Candidatus midichloria* (20,638 reads). They were found in ticks from all sampling sites, although their relative abundance varied slightly. Important differences can be observed for *Spiroplasma*, which was highly abundant in Bignoux and Lezay (7629 and 5991 reads, respectively), but drastically reduced in Hiesse (1538 reads) and Tonnay-Charente (447 reads). *Psychrobacter* was well represented in Bignoux and Tonnay-Charente but almost absent in Hiesse and Lezay (350 and 10 reads, respectively). According to our data, the sampling origin also impacted the relative abundance for *Anoxybacillus* and *Wolbachia*, which were well represented in Bignoux and Hiesse but only yielded few reads in Lezay and Tonnay-Charente. In contrast, *Mycobacterium*, *Rickettsiella* and *Rhodococcus* were present in Lezay and Tonnay-Charente but only scarcely detected in Bignoux and Hiesse. *Rickettsia* (4.19%) and *Borreliella* (1.76%), known to contain pathogenic species, were found at all sampling sites, although in low abundance (Figure 2). In our dataset, 48/175 (27.43%) individuals were positive for *Rickettsia*. The reads grouped in the category “other” represent between 20.70% (Bignoux) and 24.80% (Lezay) of all reads for each sampling site. This category includes 584 bacterial genera unambiguously assigned but detected in low abundance compared to the most present genera (Supplementary Materials Table S1). Among those, the dataset was enriched by the phylum Proteobacteria (33.90%) followed by Actinobacteria (19.80%) and Firmicutes (18.30%). On a geographical scale, significant variations in the abundance of bacterial genera have been highlighted, indicating that the location of the ticks influences their internal microbiota.

### 3.4. Site-Dependent Intra-Tick Diversity

**Figure 3:**
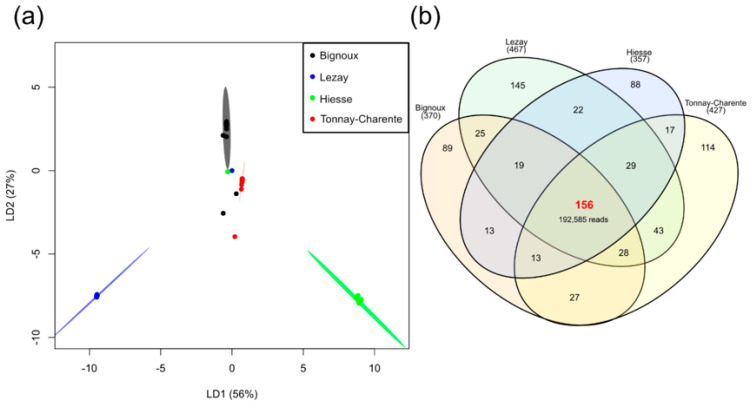
Intra-tick bacterial diversity for each site. (**a**) Site-dependent linear discriminant analysis. Each individual is represented by a single dot. Confidence ellipses were drawn with a confidence of 0.95. These axes were chosen as they depict the best separation between sampling sites; (**b**) Venn diagram of all the taxa identified. Sampling sites Bignoux, Lezay, Hiesse and Tonnay-Charente presented 370, 467, 357 and 427 different taxa, respectively.

The differences in the relative abundance of genera found according to the sampling sites raised the question of whether there is a significant difference or not when comparing the overall diversity. In an attempt to answer this question, a linear discriminant analysis was performed, and the results are shown in Figure 3a.

Almost all individuals from each sampling site clustered together, indicating a homogenous bacterial diversity in our collection. Clusters are well separated, underlining a difference in internal bacterial diversity depending on the sampling collection site. The most important difference in intra-tick bacterial diversity is observed between samples collected in Hiesse (green) and Lezay (blue), the two geographically closest sampling sites (Figure 1). Clusters for Bignoux (black) and Tonnay-Charente (red), although geographically distant, are close to each other. This indicates only little variation in the overall bacterial composition in nymphs from these two sites. Thus, the geographical distance might not be the only parameter to consider when explaining intra-tick bacterial diversity.

### 3.5. Individual Nymph Internal Core Microbiota

**Figure 4:**
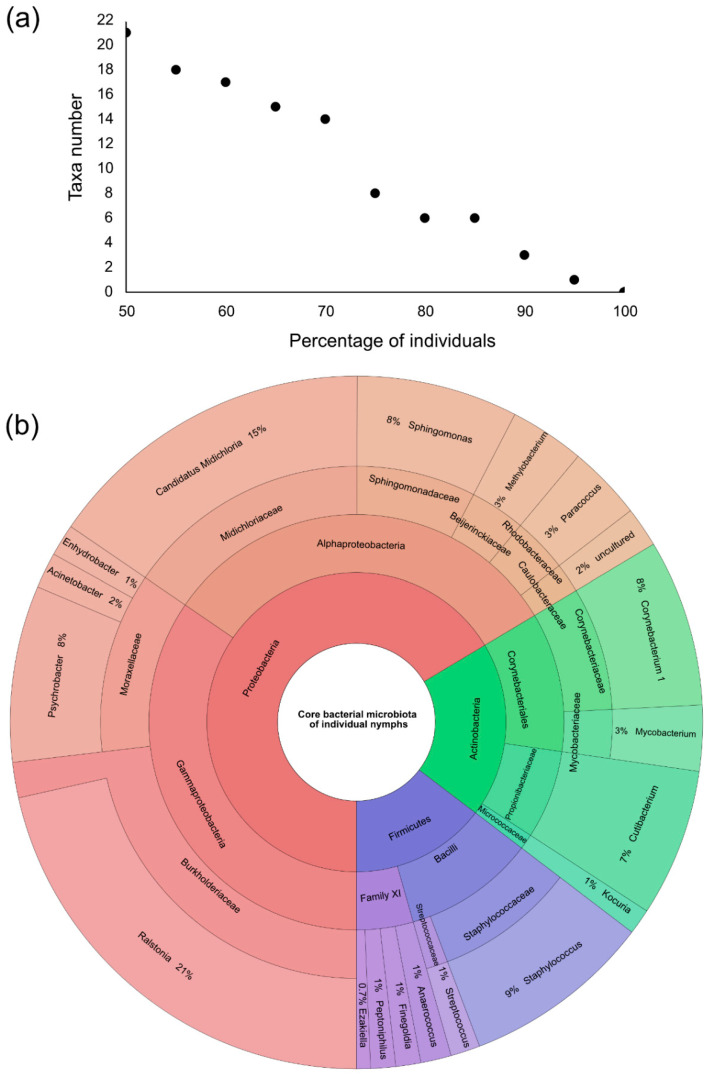
Core internal bacterial microbiota of individual nymphs. (**a**) The number of taxa present in nymphs. Each black dot represents the number of taxa present in the corresponding percentage of individuals. Twenty-one taxa were present in at least 50% of the collected individuals; (**b**) Krona chart representation showing the twenty-one taxa present in at least 50% of collected nymphs. These taxa represent the internal core microbiota at the individual level.

In an attempt to determine how many taxa were present in all geographical sites and to define a potential internal core microbiota, a Venn diagram was generated (Figure 3b). A total of 156 taxa (including the twenty most abundant) were present in at least one individual from each sampling site. These taxa represented almost all the reads, i.e., 94.47% (192,585/203,857 reads). The taxa composing this core microbiota and their relative abundance in the dataset are presented as a Krona chart (Supplementary Materials Figure S4). To go further, a core microbiota at the individual level was defined using the core-features function implemented in QIIME 2. The number of taxa present in 50–100% of the individuals was calculated (Figure 4a). Twenty-one taxa were present in at least 50% of nymphs studied; this number dropped to eight taxa when considering 75% of nymphs, and only one taxon (*Ralstonia* spp.) was present in at least 95% of the individuals. None of our taxa were present in 100% of the collected nymphs. The twenty-one taxa present in 50% of the nymphs accounted for 52.67% of all reads (107,369/203,857). Of these twenty-one taxa, twelve were part of the twenty most abundant taxa (Figure 4b). Others (*Anoxybacillus*, *Borreliella*, *Mycobacterium*, *Rhodococcus*, *Rickettsia*, *Rickettsiella*, *Spiroplasma* and *Wolbachia*) were present in high abundance but not evenly represented among samples. This was due to a high relative abundance of these bacteria in only some *I. ricinus* nymphs. These results highlighted the bacterial heterogeneity that can occur at the individual scale, especially regarding genera containing pathogenic species (*Borreliella* and *Rickettsia*).

### 3.6. Borreliella Prevalence and Diversity

**Figure 5:**
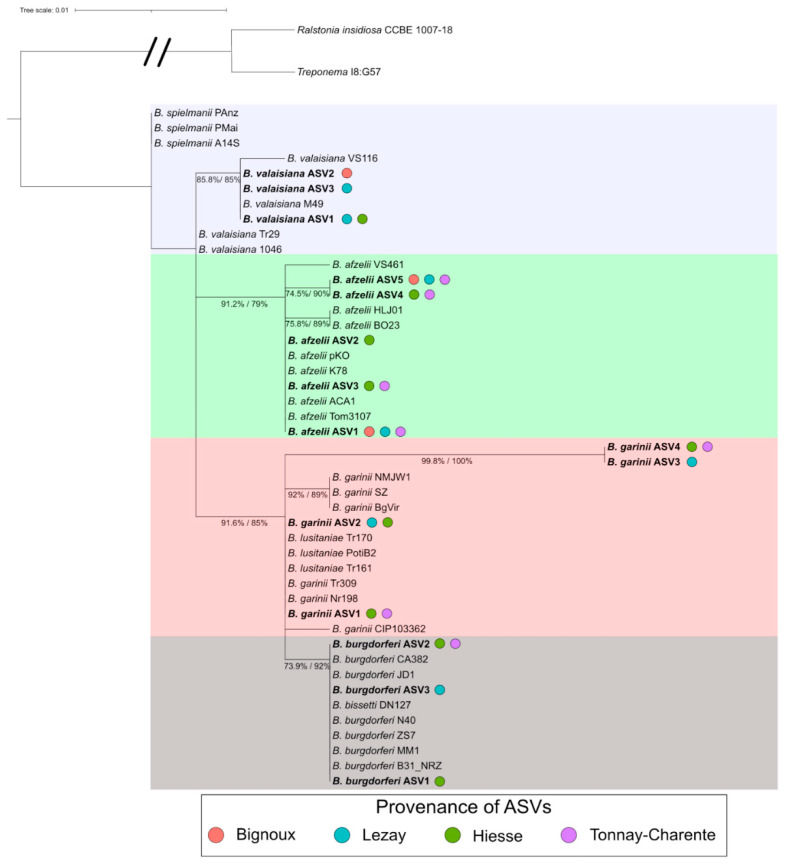
Phylogenetic tree of the *Borreliella* assigned ASVs. The tree was generated using a Kimura Two-Parameter substitution model, allowing for a proportion of invariable sites (K2P + I) model. Arbitrary colors were added to separate the four clades generated from this analysis. Colored circles indicate in which sampling sites each ASVs was found. Percentages indicate the results from the SH-like approximate likelihood ratio test and bootstraps, respectively.

Among what could be defined as a core internal microbiota in this study, some bacterial genera containing TBPs were found. Out of the TBPs that can be transmitted by the bite of *I. ricinus* nymphs, bacteria of the genus *Borreliella* remain the most monitored. In our dataset, this genus was part of the most represented genera (see Figure 2), with 3582/203,857 (1.76%) reads obtained. The reads were recovered from ticks sampled at all sites, and the total number of reads differed only slightly between sites (from 702 reads in Lezay to 1094 reads in Hiesse). To further explore the taxonomic affiliation of *Borreliella* ASV, a phylogenetic analysis was carried out to assess if ASVs could be assigned to a single species. Four clusters, including one or two *Borreliella* genospecies, have emerged (Figure 5). These clusters were composed of the following genospecies: *B. spielmanii* and *B. valaisiana* (in blue), *B. afzelii* (in green), *B. garinii* and *B. lusitaniae* (in red), *B. burgdorferi* and *B. bissetti* (in grey). Only *B. garinii* CIP103362 did not group correctly with other *B. garinii* partial 16S sequences. Even if this method did not allow us to accurately predict to which species an ASV belonged, differences were sufficient to assign our ASVs to one of the four clusters previously described. Thus, assumptions can be made regarding the presence and relative abundance of each *Borreliella* genospecies as well as co-detection depending on the sampling site.

**Figure 6:**
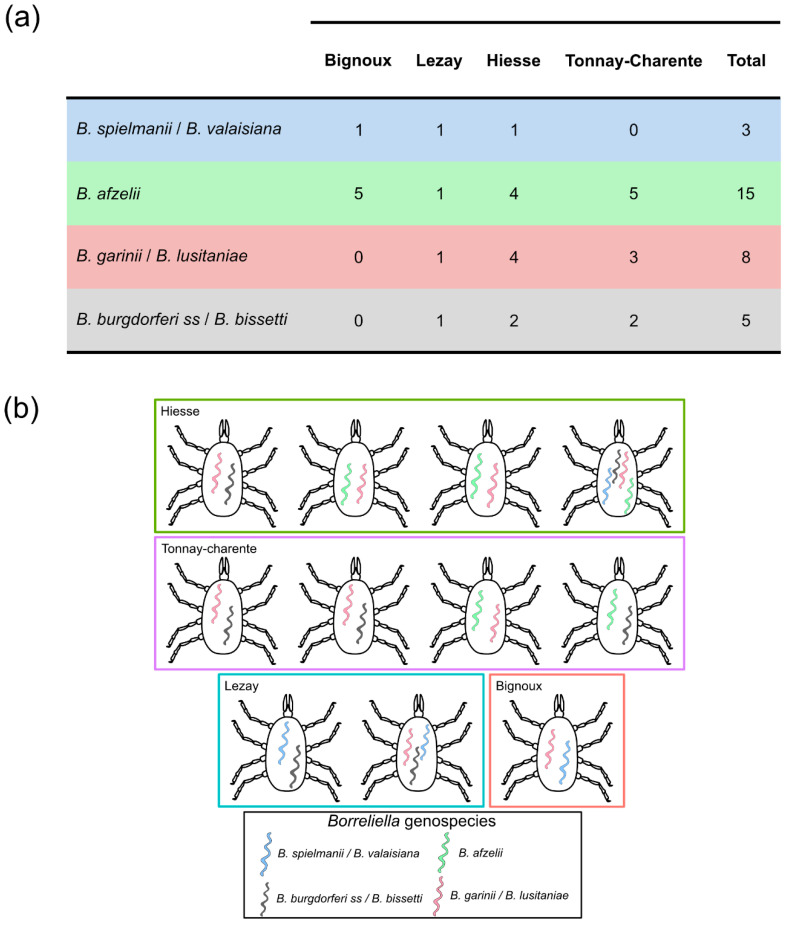
The presence of *Borreliella* in *I. ricinus* nymphs at the individual level. (**a**) The number of samples infected by *Borreliella* genospecies for each sampling site. A sample was considered positive if at least 10 reads for a genospecies were detected. Positive samples presented here can be co-infected by two or more genospecies; (**b**). Schematic representation of the different *Borrelellia* clusters found within the same infected nymph.

Overall, 11.4% (20/175) of the *I. ricinus* questing nymphs yielded 10 or more reads assigned to *Borreliella*. The most prevalent genospecies detected in our dataset was *B. afzelii,* with 15 infected nymphs and a total of 2140 reads (Figure 6a). Others genospecies clusters had fewer read counts with 561, 593 and 515 reads for *B. burgdorferi ss/B. bissetti*, *B. garinii/B. lusitaniae* and *B. spielmanii*/*B. valaisiana*, respectively.

As sequencing of individual nymph bacterial microbiota can yield several distinctive *Borreliella* ASVs, the term co-detection will presently be used when ASVs from at least two different genospecies were retrieved in a single individual. Out of the 20 nymphs positive for *Borreliella*, 11 exhibited a co-detection of at least two genospecies (Figure 6b). The most frequent co-detection was between *B. afzelii* and *B. garinii*/*B. lusitaniae* (3/11) together with *B. burgdorferi ss/B. bissetti* and *B. garinii/B. lusitaniae* (3/11). Other co-detections were between *B. afzelii* and *B. spielmanii*/*B. valaisiana* (Bignoux), *B. burgdorferi ss*/*B. bissetti* and *B. spielmanii*/*B. valaisiana* (Lezay) and *B. afzelii* and *B. burgdorferi ss*/*B. bissetti* (Tonnay-Charente). One case of triple detection was identified in Lezay: *B. spielmanii*/*B.* valaisiana, *B. garinii*/*B. lusitaniae* and *B. burgdorferi ss/B. bissetti*. Similarly, the four genospecies were detected in a single individual in Hiesse (Figure 6b).

Two or more *Borreliella* genospecies were present in 55% of *Borreliella* positive samples, and 6.3% of all the samples studied.

## 4. Discussion

Ticks are blood-sucking arthropods known to host a wide microbial diversity, including symbionts and pathogens. In this study, a focus was made on *I. ricinus*, which is the primary vector of *Borreliella*, the bacteria responsible for LD [40,43]. These bacteria could be acquired through feeding on infected wild animals between each developmental stage or through co-feeding [3,4]. They then integrate the internal microbiota composed of commensals and symbiotic microorganisms as well as other pathogens potentially [5,17].

Almost all collected ticks were identified as *I. ricinus,* which is in good agreement with previous studies conducted in France [32,34,78]. The number of adult ticks (*n* = 29) represented 9.2% of our overall collection which was much lower than the number of nymphs collected (*n* = 315). This is in line with other studies that have employed a dragging protocol to collect questing ticks [78,79,80]. We chose to study *I. ricinus* nymphs only for three main reasons. Firstly, when collecting questing ticks using dragging, which is one of the most commonly used methods, the number of nymphs collected has always been reported higher than the number of adults [78,81,82]. Secondly, even though depending on many factors, researchers pointed that there are more chances to get bitten by *I. ricinus* nymphs rather than by adults at recreational sites [83]. Finally, nymphs were also described to be the principal vectors that transmit *Borreliella* to humans in Europe [84]. This analysis was performed at the individual level to also determine the importance of inter-individual variations and to investigate whether it is possible to define a core microbiota. Studies regarding the total microbial composition in ticks are scarce. In particular, many studies have focused on the detection of pathogens by molecular methods such as PCR and qPCR [32,33,78,85]. These methods, while robust, do not detect total bacterial diversity inside ticks. With the expansion and improvement of sequencing methods at lower costs, systematic sequencing of samples might be an alternative to study the total microbiota rather than focusing on pathogens only. This is especially important since numerous microbial interactions have been proven to occur inside the tick gut [5,17].

To access the internal microbiota of these ticks by metabarcoding 16S, several elements had to be taken into consideration. Intra-nymph microbiota analysis is challenging due to the low biomass of the samples. In such samples, contaminants can account for a large portion of all sequences if not treated carefully [86]. It is also necessary to get rid of the contamination introduced by all the elements associated with the cuticle, such as external flora and DNA. A recent bleach-based cuticular disinfection was shown to be efficient in removing cuticular bacterial DNA without impacting the internal bacterial diversity hosted by ticks [50]. The number of sequences for each sample is also drastically reduced when compared to ethanol-based disinfection as only internal microbiota is kept. As recently underlined, all this information raises questions about the real intra-tick bacterial diversity [28,86].

From 826,697 reads from 180 individuals, we dropped to 203,857 (175 individuals) after quality analysis, which is an average of 1165 reads per individual. It is important to point out that ticks are arthropods harboring not only bacteria but also protists and parasitoids that can themselves be infected by bacteria. It has been shown that genera such as *Wolbachia* and *Arsenophonus* detected in ticks arise most likely from parasitoid infection rather than belonging to the tick microbiota itself [87,88]. In this study, reads assigned to *Wolbachia* and *Arsenophonus* represent 1.40% and 0.76% of the total, respectively.

A core microbiota was defined at the sampling site scale. It was composed of 156 taxa and accounted for 192,585/203,857 reads (94.47%). In this core microbiota, an important number of reads (149,385) were assigned to only twenty taxa, of which the most abundant is *Ralstonia* spp. The presence of *Ralstonia* spp. in ticks has already been documented but remains scarce in literature [21,89,90]. This genus contains species omnipresent in soils and humid environments [91]. Therefore, they can be in contact with ticks during their natural cycle. However, it is surprising to find them at such a high abundance within our samples, given that the bleach-based cuticle disinfection protocol allows the removal of external bacteria and DNA. *Candidatus Midichloria mitochondrii* is the second most prevalent genera identified in our samples. It has been described as an intramitochondrial endosymbiont infecting *I. ricinus* females [92]. This bacterium is vertically transmitted to the offspring [93], which explains the high abundance found in individual nymphs. The roles played by this bacterium are still unknown, yet several hypotheses are considered [28,94]. In 2018, a study highlighted the role of *C. Midichloria mitochondrii* in the growth of *Rickettsia parkeri* (a pathogenic species of *Rickettsia*) in *Amblyomma maculatum* [95]. It must be noted that *C. Midichloria mitochondrii* is not as prevalent in *A. maculatum* as it is in *I. ricinus*. Both *Ralstonia* spp. and *C. Midichloria mitochondrii* are also members of the individual core bacterial microbiota defined in this study. This internal core microbiota is composed of twenty-one taxa present in at least 50% of nymphs. However, it is challenging to define a universal core bacterial microbiota in ticks since its composition relies on numerous factors.

Among the sequences assigned to the most abundant genera, high percentages of sequences were assigned to *Borreliella* (1.76%) and *Rickettsia* (4.19%), bacterial genera known to contain pathogenic species. A total of 603 bacterial genera were unambiguously identified in the dataset, indicating that the *I. ricinus* internal microbiota contains a few abundant bacterial genera associated with a highly diverse but poorly represented bacterial community. Interestingly, reads assigned to the genus *Legionella* were also retrieved in low abundance (0.06%). Only a couple of studies have observed the presence of reads belonging to this genus in tick samples. One is a metagenomic analysis of the pathobiome of *Haemaphysalis longicornis* (adults, nymphs and larvae) collected on Staten Island in the United States [96], and the other is a pyrosequencing analysis of the microbiota of *Hyalomma* [97]. This is of interest since several species are known to be pathogenic to humans [98]. Moreover, these bacteria and their natural vectors (phagotrophic protists) are ubiquitous in humid soils and thus can share a common habitat with ticks that are sensitive to desiccation and require an important hygrometry [99]. It is not yet possible to tell whether ticks are able to vectorize the protist and/or *Legionella* or not. In our study, 27.43% of individuals were positive for *Rickettsia*. Prevalence rates of *Rickettsia* positive ticks reported in the literature are variable. Overall, the rate retrieved in our study seems higher than the prevalence reported in other studies [100,101]. In general, *Rickettsia* detection is carried out using specific molecular amplification by PCR and qPCR. Such targeted methods can limit the detection of certain species of *Rickettsia*, resulting in a lower prevalence rate. On the contrary, using global amplification as conducted in this study can induce false positives (especially for individuals harboring few reads assigned to *Rickettsia*) and thus overestimating the prevalence rate.

In this study, a specific focus was made on *Borreliella* since LD is the most widespread vector-borne disease in the Northern Hemisphere. Here, 20 nymphs (11.4%) were positive for *Borreliella*. A phylogenetic analysis, including all 16S rRNA coding gene reads from reference strains of *Borreliella,* allowed the assignment of ASVs to one of the four clusters consisting of one or two genospecies. The most prevalent genospecies found in this study was *B. afzelii,* with 15 infected nymphs (8.57%). *B. burgdorferi ss/B. bissetti*, *B. garinii/B. lusitaniae* and *B. spielmanii*/*B. valaisiana* were the others genospecies identified but in lower abundance. Numerous studies about *I. ricinus* infected by *Borreliella* have been carried out over the last decades, generating hundreds of scientific articles on the matter with thousands of ticks analyzed. Several meta-analysis studies have been published, compiling published data over a 34 years period (1984–2017) [48,102,103,104]. It clearly appears that the infection rate of ticks depends on the life stage considered. For nymphs, the infection rate was between 10.8% [104] and 11.8% [48], which is consistent with the infection rate found in our study. Our data are also in agreement with previous studies highlighting a *B. afzelii* infection rate of around 9% in field-collected *I. ricinus* (both adults and nymphs) [32,33]. Moreover, the most prevalent genospecies in Europe are *B. afzelii*, *B. garinii* and *B. valaisiana* [102], three of the four genera retrieved in our dataset. Co-infection of *I. ricinus* with several bacteria, whether they are pathogenic or not, is a field of growing interest since the emergence of the pathobiome concept [105]. Ticks harbor a wide diversity of microorganisms, legitimating the fact that they can vectorize several pathogens at the same time. This could represent health concerns in the context of co-transmissions to humans. Here, reads assigned to at least two different *Borreliella* clusters were found in 11 individuals (11/20; 55%). *B. afzelii* and *B. garinii*/*B. lusitaniae* together with *B. burgdorferi ss/B. bissetti* and *B. garinii/B. lusitaniae* were the most co-detected partners (3/11 each). In addition, one case of triple co-detection and one case of quadruple co-detection were observed. Few studies focusing on pathogens detection in *I. ricinus* have found high rates of co-infection with at least two pathogens [32,33]. Among them, co-infections with at least two species of *Borreliella* were the most prevalent, especially between *B. afzelii* and *B. garinii*. It should be stressed that adult ticks were included in both studies, so they have an extra blood meal and therefore more chances to carry pathogens. This could be an explanation for the higher co-infection prevalence they describe. However, co-detections of several *Borreliella* genospecies in nymphs are intriguing. Studies about *Borreliella* infection in animals have previously highlighted preferential hosts depending on the genospecies [106,107]. *B. garinii* is commonly found in birds, whereas *B. afzelii* is more commonly detected in rodents. However, co-infections by two or more *Borreliella* in nymphs are contradictory to these results. Since questing nymphs only had one blood meal, if co-infections are detected at this life stage, they most likely arise from a co-infection in the host on which the blood meal was taken or by co-feeding. The latest has been proven to be a direct tick-to-tick mode of bacterial transmission [4]. If larvae feed alongside already infected ticks, they can be infected with multiple pathogens during a single blood meal, resulting in co-infections in the nymphal stage.

## 5. Conclusions

To conclude, this study, carried out on nearly 200 nymphs collected at different locations, allowed us to describe the internal bacterial microbiota of each individual and also to define a core microbiota of 156 taxa. The inter-individual variations observed depend only slightly on the sampling site. Finally, one nymph out of ten collected carried *Borreliella* sp. At least two *Borreliella* genospecies were also co-detected in 55% of infected nymphs, which raises the question of interactions between these bacteria but also of their potential co-transmission to a host. To go further, studies characterizing intra-tick tissue tropism of the co-detected *Borreliella* strains should be carried out.

## Figures and Tables

**Table 1:** Diversity index calculated for each sampling site. Calculations were carried out after probable contaminant reads removal. An uneven number of individuals is due to samples exclusion based on the Bray-Curtis dissimilarity index calculation.

Sampling Site	Number of Individuals	Average Reads per Tick	Shannon H Index (±SD ^1^)	Shannon E Index (±SD ^1^)	Observed ASVs
Bignoux	45	1055	2.83 (±0.81)	0.77 (±0.15)	1079
Lezay	43	1167	3.25 (±0.71)	0.76 (±0.13)	1371
Hiesse	43	1043	2.90 (±0.78)	0.73 (±0.14)	1019
Tonnay-Charente	44	1394	3.21 (±0.80)	0.75 (±0.15)	1433

^1^ SD: Standard Deviation.

## Data Availability

The data analyzed in this study are publicly available in the Sequence Read archive of the NCBI under the BioProject ID PRJNA727936.

## Supplementary Materials

The following are available online at https://www.mdpi.com/article/10.3390/microorganisms9071526/s1, Figure S1. Dendrogram drawn based on the Bray-Curtis dissimilarity index. Bacterial diversity in samples written in red most likely arise from experimental contaminants. These samples were removed from the dataset for further analysis; Figure S2. Rarefaction curves of all individuals based on (a) number of observed ASVs; (b) Shannon H index. All samples are represented as a single bar and colored based on the sampling site. All curves reached a plateau for sequencing depths higher than 2500 reads; Figure S3. Violin plot of alpha diversity metrics. (a) Shannon H index; (b) Shannon evenness index; (c) Number of observed ASVs. All individuals from each sampling site were included to generate the violin plots; Figure S4. Krona chart of the core microbiota defined in this study. The diagram was generated using the 156 taxa found at least once at each sampling site. The relative abundance of each taxa was calculated by dividing the number of reads affiliated to each taxon by the total number of reads (203,857) and are presented as percentages.
