# Supplementary material for: Exploring the Individual Bacterial Microbiota of Questing Ixodes ricinus Nymphs
Source: Microorganisms. 2021 Jul 17;9(7):1526. doi: 10.3390/microorganisms9071526 (PMC8303981; doi:10.3390/microorganisms9071526)
Supplement: Supplementary file 1 [file microorganisms-09-01526-s001.zip › microorganisms-1262277-supplementary.pdf]

Exploring the individual bacterial microbiota of questing *Ixodes ricinus* nymphs.

Aurélien ALAFACI, Alexandre CREPIN, Sabine BEAUBERT, Jean-Marc BERJEAUD, Vincent DELAFONT* and Julien VERDON*✉

Laboratoire Ecologie & Biologie des Interactions, UMR CNRS 7267, Université de Poitiers, 1 Rue Georges Bonnet, TSA 51106, 86073 POITIERS Cedex 9, France
* Co-last authors
* Correspondence: E-mail: julien.verdon@univ-poitiers.fr Phone: +33 5 49 45 36 93

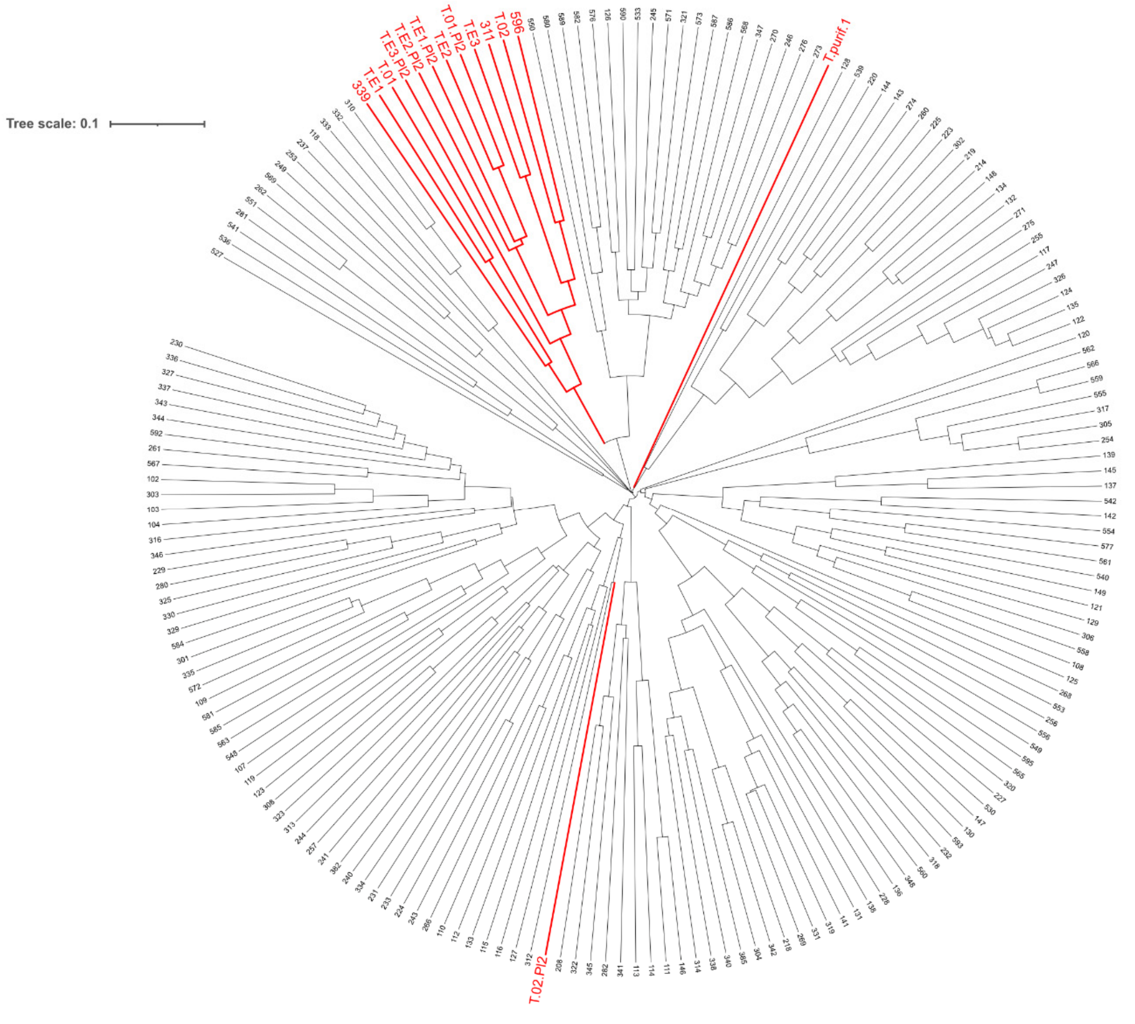

Figure S1. Dendrogram drawn based on the Bray-Curtis dissimilarity index. Bacterial diversity in samples written in red most likely arise from experimental contaminants. These samples were removed from the dataset for further analysis.

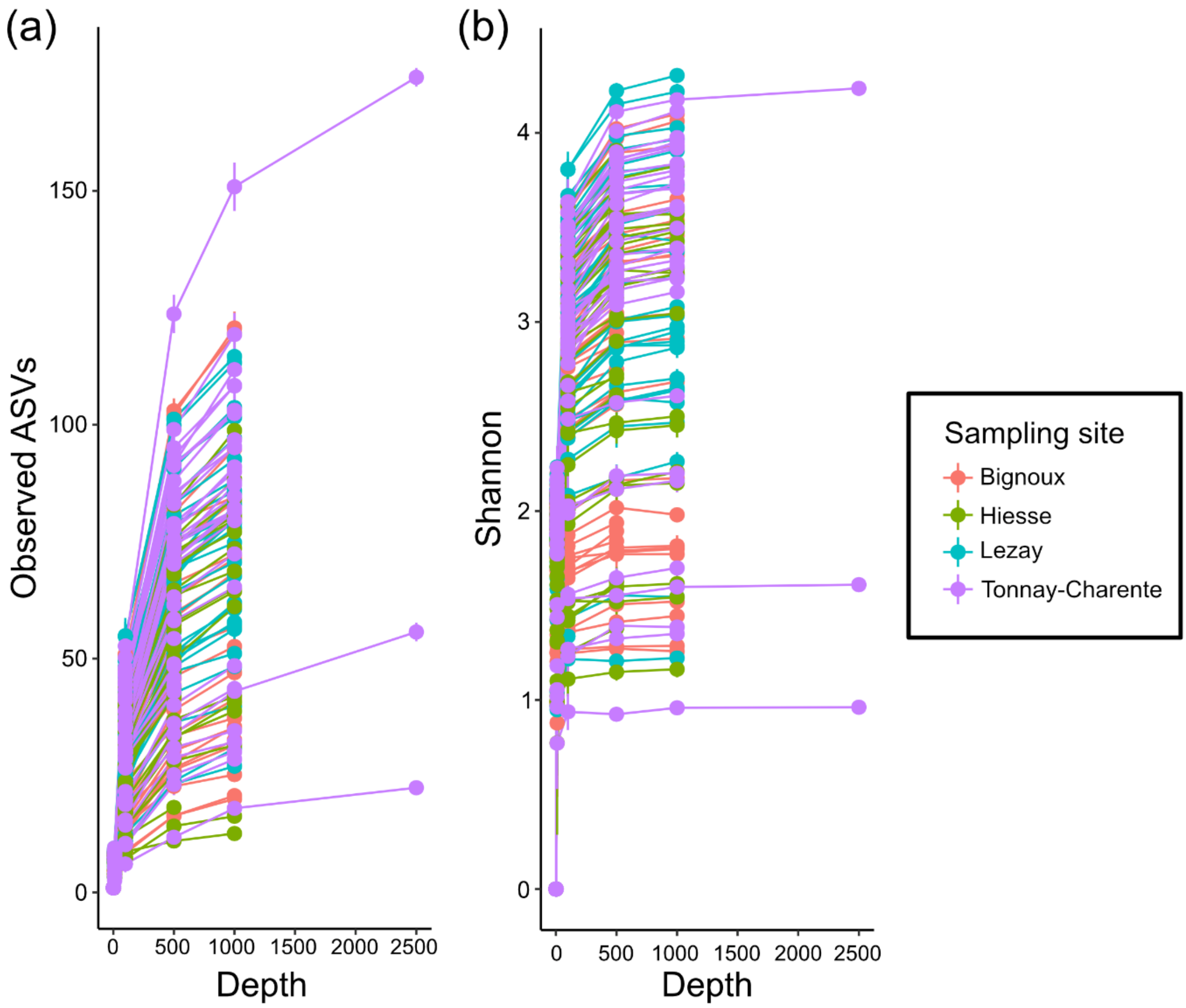

Figure S2. Rarefaction curves of all individuals based on **(a)** number of observed ASVs; **(b)** Shannon H index. All samples are represented as a single bar and colored based on the sampling site. All curves reached a plateau for sequencing depths higher than 2500 reads.

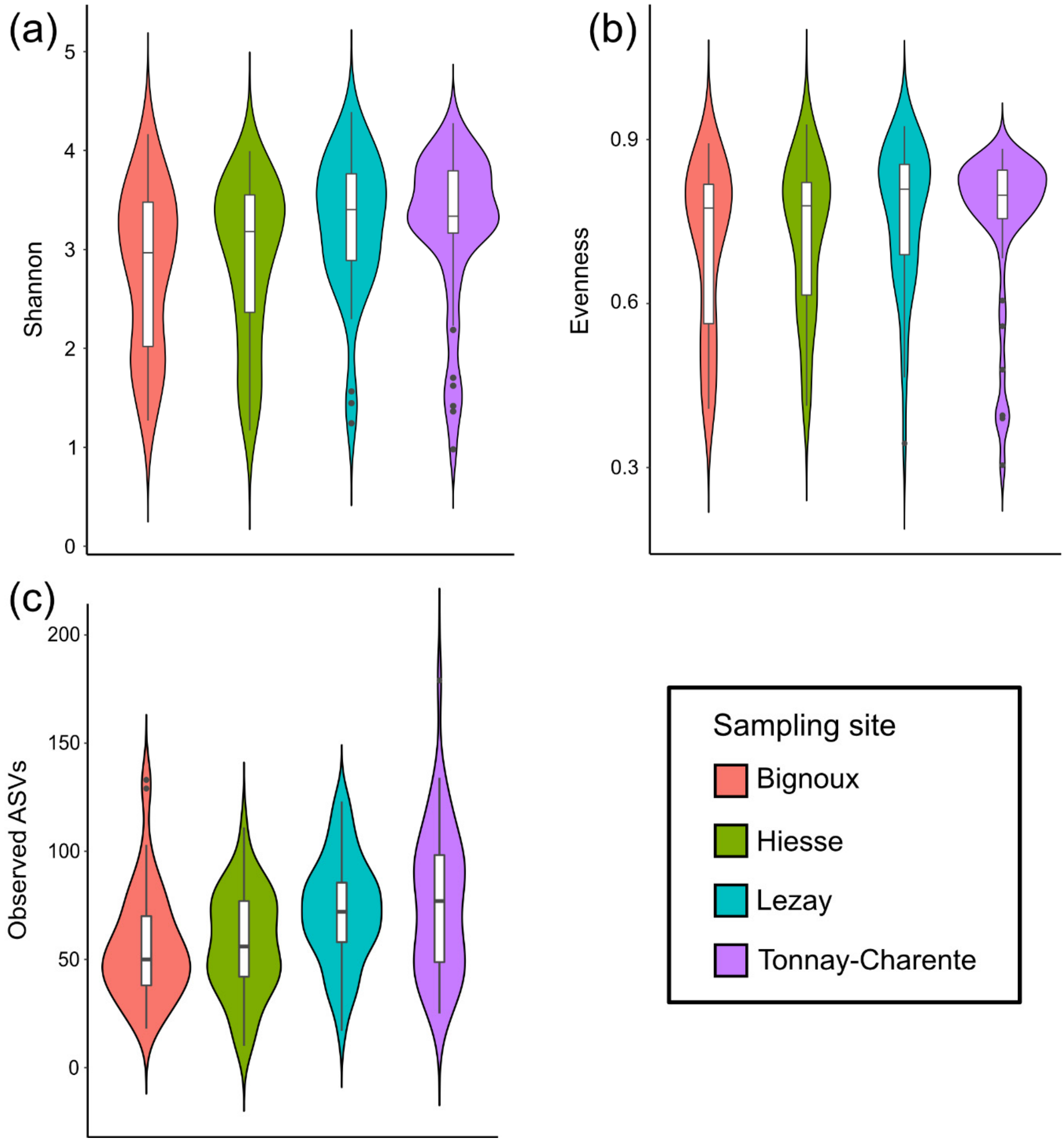

Figure S3. Violin plot of alpha diversity metrics. **(a)** Shannon H index; **(b)** Shannon evenness index; **(c)** Number of observed ASVs. All individuals from each sampling site were included to generate the violin plots.

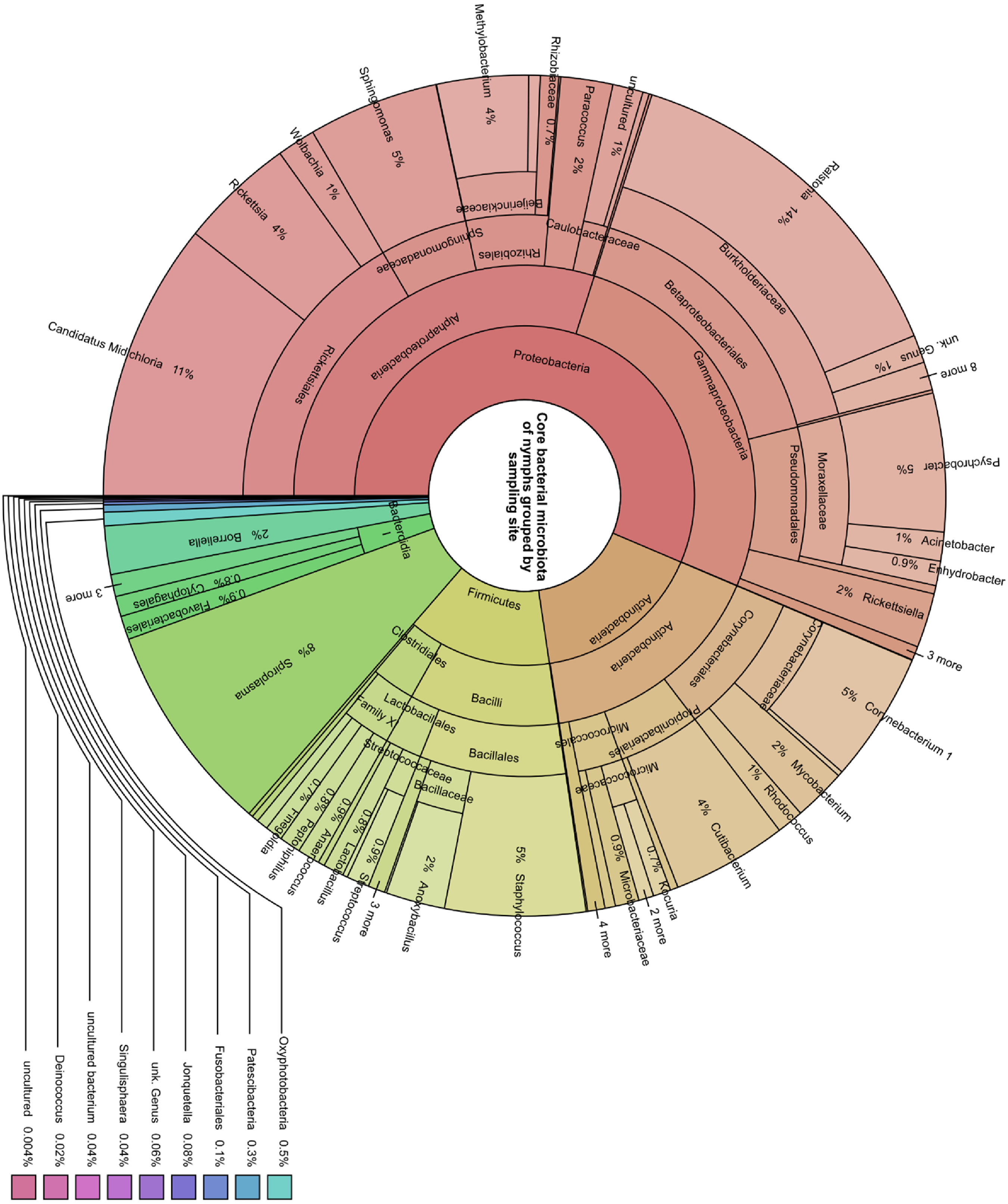

Figure S4. Krona chart of the core microbiota defined in this study. The diagram was generated using the 156 taxa found at least once at each sampling site. The relative abundance of each taxa was calculated by dividing the number of reads affiliated to each taxon by the total number of reads (203,857) and are presented as percentages.
